# Consumption of milk and dairy products and risk of asthma in children: a systematic review and Meta-analysis

**DOI:** 10.1186/s13690-023-01162-8

**Published:** 2023-08-16

**Authors:** Feng Song, Yang Xie, Nannan Guo, Hulei Zhao

**Affiliations:** 1 Collaborative Innovation Center for Respiratory Disease Diagnosis and Treatment & Chinese Medicine Development of Henan Province, Henan University of Chinese Medicine, Henan, China; 2 Henan Key Laboratory of Chinese Medicine for Respiratory Disease, Henan University of Chinese Medicine, Henan, China; 3 Department of Respiratory Diseases, The First Affiliated Hospital of Henan University of Chinese Medicine, Henan, China

**Keywords:** Milk, Dairy, Asthma, Meta-analysis, Systematic review

## Abstract

**Background:**

Some reports demonstrate that asthma benefits from milk and dairy products, however, the findings are controversial. We used meta-analysis as a tool to summarize published data on the association between dairy products consumption and asthma.

**Methods:**

A systematic literature search was conducted to identify studies of dairy products and asthma in children in PubMed, ISI (Web of Science), and EMBASE until 21 July 2022. Random-effect meta-analyses with summarized data were performed for total (high/low) milk and dairy intake. Subgroup analysis was used to identify sources of variation in responses. Publication bias and sensitivity analysis were done to examine the stability of results.

**Results:**

There was no correlation between consumption of dairy products and reduced risk of asthma (OR = 0.82; 95% CI: 0.60–1.05). Our results revealed that elevated consumption of milk and dairy has significant correlation with reduced risk of asthma in Non-Asian population (OR = 0.74; 95% CI = 0.51–0.96) and high quality studies (OR = 0.73; 95% CI = 0.50–0.95). No individual study influence and publication bias was seen in the sensitivity analysis and publication bias assessment.

**Conclusion:**

There was no correlation between consumption of dairy products and reduced risk of asthma. However, we observed that elevated consumption of milk and dairy has significant correlation with reduced risk of asthma in Non-Asian population and high quality studies. More high-quality and population-specific studies should be conducted to determine the risk link between milk consumption and asthma in children.

**Supplementary Information:**

The online version contains supplementary material available at 10.1186/s13690-023-01162-8.

## Background

The dramatic increase in noncommunicable diseases (NCD) prevalence can be explained by the emergence of risk factors or the loss of protective factors, or both in the past decades [1]. As the most frequent NCD in children, asthma has become a heavy burden on public health in high-income countries and urbanized areas of low-income and middle-income countries [2–4]. Given the high prevalence asthma, further exploration of specific risk factors associated with the disease may help develop better prevention and therapeutic treatment measures.

There is growing evidences that diet plays a big role in the development and treatment of asthma [5]. Diet, a modifiable factor, can be essential to decrease incidence of asthma in primary preventive measures [6]. Milk and dairy products with unique micronutrient combinations have been recommended in most dietary guidelines around the world. Previous systematic review and meta-analysis indicated a protective role of the Mediterranean diet against childhood asthma [7, 8]. Several observational studies suggest that drinking milk may have a protective effect on children with asthma, but the role is controversial in children [9–15]. A cohort study demonstrates 31% reduced risk of asthma by consuming full cream milk daily [9]. In girls, infrequent milk consumption increases 11% risk of asthma in a nested case-control study [10]. Yang et al. [11] and Feng et al. [12] found 60% and 68% increased risk of asthma by high consumption of milk. In addition, some others observed no a significant association [13–15]. Therefore, we conducted a systematic review and meta-analysis to determine the impact of milk and dairy consumption and the risk of childhood asthma.

## Methods

### Search strategy

Our study was conducted by using systematic literature search in PubMed/Medline, EMBASE and ISI web of Science, prior to 21 July 2022 based on the PRISMA (Preferred Reporting Items for Systematic Reviews and Meta-analyses) statement. The detail search strategy is shown in the Supplemental Table S1. To avoid less precise and reduce possibly biased effects, the reference lists of articles retrieved by this study were also checked to include all the published data.

### Inclusion criteria

In the present meta-analysis, two investigators were asked to independently select articles based on three certain criteria as follows: (1) all observational studies (cohort, case-control or cross-sectional) performed on children who were younger than 18 years old; (2) studies used explicit method for assessing milk and dairy consumption and asthma diagnosis; (3) articles reported odds ratios (ORs), hazard risks (HRs), or relative risks (RRs) accompanied by 95% confidence intervals for asthma or data which can be calculated for confidence intervals, (4) the language is limited to English. Discrepancies were resolved by discussion with a third investigator.

### Exclusion criteria

Meta-analyses, reviews, letters, and comments were excluded. When multiple publications were available for the same study, only the most comprehensive publication was included in the present meta-analysis.

### Data extraction

Information was independently extracted by two investigators for all the included articles as follows: publication time, study design, first author, country, number of cases, sample size, age range, gender, exposure variable, assessment of exposure, outcome variable, assessment of outcome, relevant effect sizes that are aORs, aRRs, or aHRs with their confidence intervals (95%), and adjusted covariates.

### Quality assessment

The Newcastle–Ottawa Scale (NOS) was used to assess the qualities of cohort and case-control studies included. The NOS assigns up to 9 points per study: 2 points for comparability, 4 points for selection, and another 3 points for assessment of exposures and outcomes. For the analysis in this study, we calculated the sum of each item score, with scores of seven and above representing high quality and scores below seven representing low quality.

### Statistical analysis

The term ‘‘OR’’ was used as a generic term for ORs and HRs in the study. Based on the OR of asthma for the lowest vs. the highest intake of dairy products reported in one publication [10], we computed the inverse OR and its upper and lower limits. There are several different ORs of asthma reported by two studies [13, 16] for various categories and levels of dairy consumption. Those ORs were integrated in a preliminary meta-analysis by using a fixed-effects model and pooled for each study. Overall effect size was then calculated for all included studies by using a random-effects model which takes into consideration of between-study variance. The detailed data was presented using the forest plot. *I*^*2*^ index measure and Cochran’s Q test were conducted to evaluate heterogeneity. *P* < 0.05 was identified being statistically significant for the Q statistic; for the *I*^*2*^ statistic, *I*^*2*^ values of approximately 75, 50, and 25% refer to high, moderate, and low heterogeneity, respectively. We used subgroup analysis to explore possible sources of heterogeneity in the study, and sensitivity analysis to assess the level to which inferences have the possibility to depend on one particular study. Egger’s regression asymmetry test and visual inspection of Begg’s funnel plots were used to examine publication bias in this study. The software, STATA version 14.2 (STATA Corp., College Station, Texas, USA) was used as a tool for statistical analyses, and *P*-value qualities < 0.05 were considered as statistically significant.

## Results

### Study features and literature-search results

**Fig. 1:**
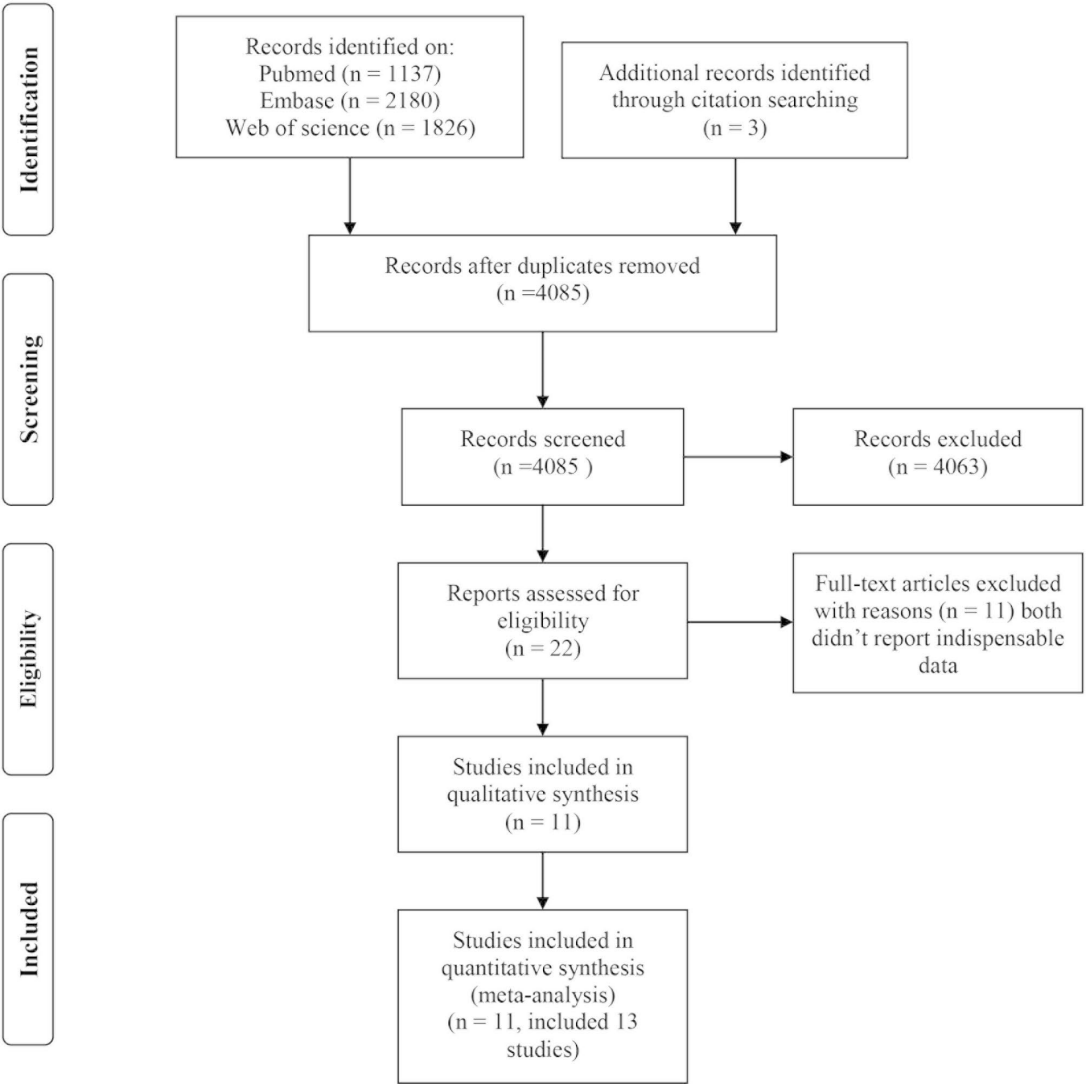
The flow diagram of study selection

There were 11 articles [9–19] (13 independent studies included) which were published between 2000 and 2022, identified for our meta-analysis (Fig. 1). Of the included articles, 3 were cohort studies, 4 were of cross-sectional design, and 4 were of case-control design. The basic features of those studies included in our meta-analysis were shown in Supplementary Table S2.

### Findings from meta-analysis

**Fig. 2:**
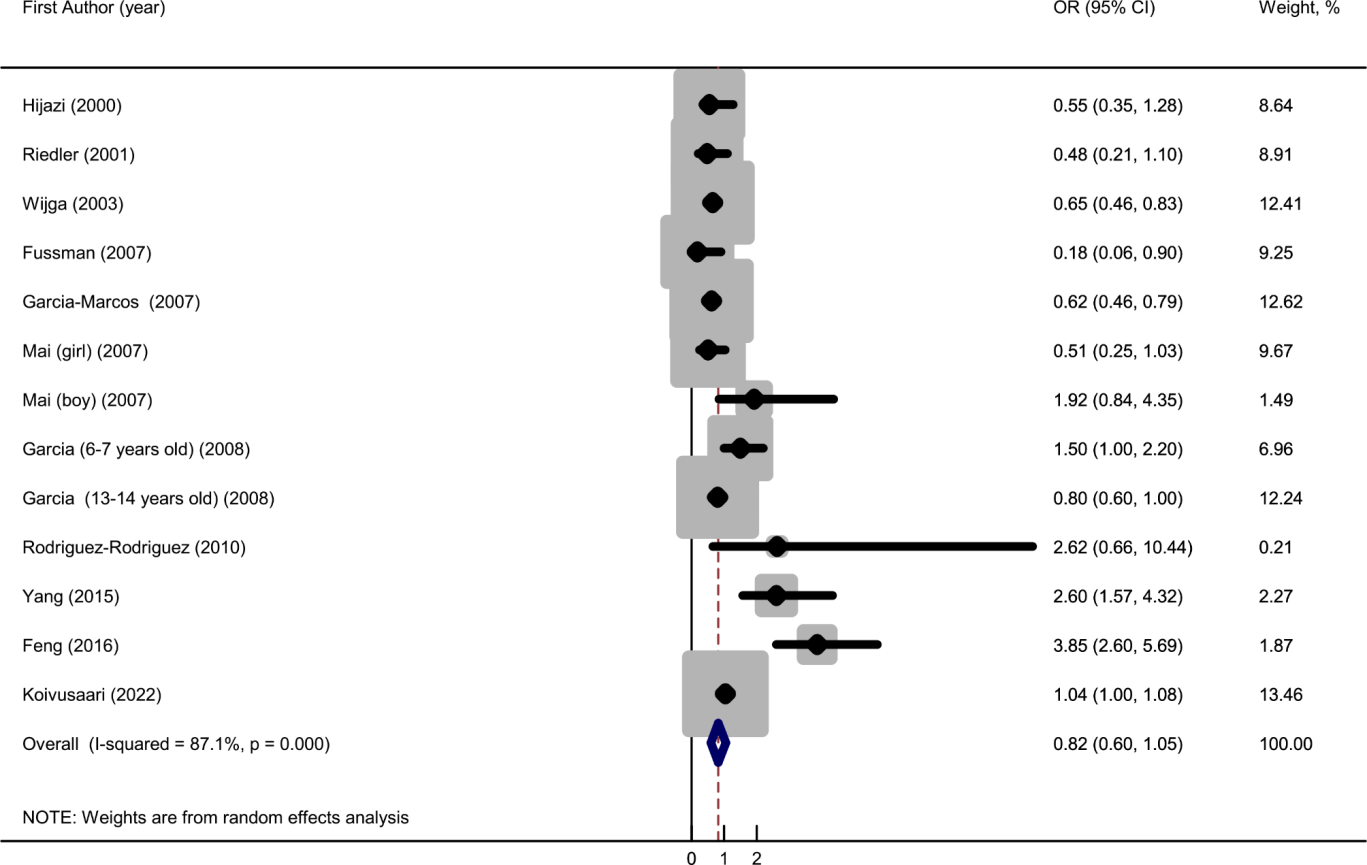
Forest plots of the association between milk and dairy consumption and risk of asthma

The summary OR of high vs. low milk and dairy consumption on asthma was 0.82 (95% CI: 0.60–1.05), with a high substantial heterogeneity (*I*^*2*^ = 87.1%, *P* < 0.001; Fig. 2).

### Subgroup and sensitivity analyses and publication bias

To identify the potential sources of heterogeneity and examine the robustness of the result, subgroup analysis was conducted according to study design, quality of study, sample size, location, and adjustment for gender and age (Table 1and Supplementary Figure S1). The correlation between risk of asthma and dairy products consumption was not statistically significant (OR = 0.67; 95% CI: 0.26–1.08) in cohort studies and case-control/cross-sectional designs (OR = 0.93; 95% CI: 0.63–1.24). Reduced risk of asthma was found significantly associated with greater dairy products consumption in Non-Asian population (OR = 0.74; 95% CI = 0.51–0.96) and high quality studies (OR = 0.73; 95% CI = 0.50–0.95). However, no source of between-study heterogeneity was observed. To estimate the accuracy and robustness of the pooled effect, we excluded each study separately and recalculated the combined ORs of the remaining ones for sensitivity analysis, and we found there were no excessive influences from any individual study on the pooled effect. Moreover, to detect publication bias, we used Egger test (*P* = 0.0864) and Begg’ funnel plot which were showed in Supplementary Figure S2.

**Table 1:** Results of subgroup Analysis for milk and dairy consumption and risk of asthma

	N	OR (95% CI)	*I*^*2*^(%)	*P* Heterogeneity
**All studies**	13	0.82 (0.60–1.05)	87.1	< 0.001
Study design				
Cohort	3	0.67 (0.26–1.08)	93.7	< 0.001
Case-control/cross-sectional	10	0.93 (0.63–1.24)	75.4	< 0.001
Location				
Asian countries	3	2.24 (0.12–4.36)	90.9	< 0.001
Non-Asian countries	10	0.74 (0.51–0.96)	87.3	< 0.001
Sample size				
< 1000	8	1.02 (0.48–1.57)	91.0	< 0.001
≥1000	5	0.86 (0.61–1.10)	78.3	< 0.001
Quality score				
< 7	4	2.14 (0.44–3.83)	88.0	< 0.001
≥7	9	0.73 (0.50–0.95)	88.2	< 0.001
Adjustment factors				
Gender				
Yes	8	0.72 (0.43–1.02)	90.6	< 0.001
No	5	1.12 (0.63–1.62)	73.9	0.004
Age				
Yes	4	1.33 (0.53–2.13)	84.4	< 0.001
No	9	0.71 (0.51–0.92)	68.0	0.002

## Discussion

This meta-analysis including 13 studies aimed to clarify the association between dairy products consumption and risk of asthma. The result of our study supported that greater dairy products consumption was not linked to reduced risk of asthma, however, inverse association was observed in Non-Asian population and high quality included studies.

The inconsistent association among studies might be related to inverse effect of raw milk (protective factor) and heat-treated milk (risk factor) [19]. There was no negative association between dairy products consumption and asthma was found. The reason could be that the designs of these studies often have intrinsic biases which may cause the results of these studies inaccurate in case-control and cross-sectional studies. Prospective cohort studies have better control for potential cofounders and sufficient follow-up time to identify asthma patients in general. Thus, they have the ability to provide more reliable estimates compared to cross-sectional studies. We found that greater intake of dairy and milk were not associated with reduced risk of asthma in pooled cohort studies, while an inverse association was seen in two of three included cohort studies. Due to limited number of prospective cohort studies on this issue, it seems that additional data are required to come to a definite conclusion in this regard.

Our study suggested the negative association between dairy products consumption and risk of childhood asthma in Non-Asian population but not found in Asian population. A reasonable explanation could be the limited dairy products consumption in Asian population compared to Non-Asian population. According to the findings reported by the International Study of Asthma and Allergies in Childhood (ISAAC) in 1998, the prevalence of asthma-related symptoms declined in Western Europe, but increased in Asia Pacific [20]. The power of high quality studies was greater than low quality studies in statistic. No negative association was observed in low quality studies included, which might be relative to their low power. Compared to low quality study included, high quality study included could be more really reflect actual association between the risk of developing asthma and dairy products consumption. These might explain the difference between high quality studies included and low quality studies included for this association.

Some specific biological mechanisms could illustrate the relationship between dairy products consumption and childhood asthma. Milk and dairy products are rich in proteins, oligoelements and macroelements, as well as lipophilic and hydrophilic vitamin [21]. The protective impact of dairy products intake on asthma could be explained by their effect on immune cell function, corticosteroid responsiveness, oxidative stress, and airway remodeling [22, 23].

### Strengths and Limitations

This systematic review and meta-analysis used a combination of evidence from all studies published to assess if milk and dairy consumption has association with the risk of childhood asthma, which increased the statistical power to identify a negative association of dairy products intake and risk of asthma. In addition, the potential publication bias in the present meta-analysis was minimized though the application of a broad literature search as mentioned above. Since the development of asthma symptoms could be linked to substantial factors [2, 24], the multivariate regression model was applied to adjust effects of the main influencing factors included in most of the eligible studies in this meta-analysis. Thus, the impact of other factors may be relatively limited. We could not discover potential sources of heterogeneity in included studies by various subgroup analyses. Meanwhile, there were no statistically significant publication bias and influence on the association from individual study in sensitivity analysis, due to the high-quality studies included. We could not register the protocol of the review in the associated website, but our study was conducted based on the PRISMA statement. Two investigators were asked to independently search, select, and extract relevant articles to decrease potential bias.

Considering articles included for the final analysis were limited, we did not perform linear and non-linear dose–response meta-analysis (DRMA) on the theme in this study. Future studies examining multiple aspects of milk and dairy consumption are needed to explore their association.

## Conclusion

There was no correlation between consumption of dairy products and reduced risk of asthma. However, we observed that elevated consumption of milk and dairy has significant correlation with reduced risk of asthma in Non-Asian population and high quality studies. More high-quality and population-specific studies should be conducted to determine the risk link between milk consumption and asthma in children.

### Electronic supplementary material

Below is the link to the electronic supplementary material.

Supplementary Material 1

## Data Availability

Data are available upon reasonable request. The data are available upon request from the corresponding author.

## Abbreviations

NCD: Noncommunicable diseases
CI: Confidence interval
OR: Odds ratio
HR: hazard risk
NOS: Newcastle–Ottawa Scale
ISAAC: International Study of Asthma and Allergies in Childhood
