# Supplementary material for: Consumption of milk and dairy products and risk of asthma in children: a systematic review and Meta-analysis
Source: Arch Public Health. 2023 Aug 16;81:147. doi: 10.1186/s13690-023-01162-8 (PMC10428541; doi:10.1186/s13690-023-01162-8)
Supplement: Supplementary file 1 — Supplementary Material 1 [file 13690_2023_1162_MOESM1_ESM.docx]

**Supplemental Table S1. Search strategy**

PubMed (<http://www.ncbi.nlm.nih.gov/pubmed>)

Action 1 Determinants

#1 (dairy [Title/Abstract]) OR (milk*[Title/Abstract]) OR (cheese*[Title/Abstract]) OR (yogurt*[Title/Abstract]) OR (yogurt*[Title/Abstract]) OR (butter [Title/Abstract]) OR (buttermilk [Title/Abstract]) OR (dietary pattern*[Title/Abstract]) OR (dairy products [MeSH Terms]) OR (milk [MeSH Terms]) OR (cheese [MeSH Terms]) OR (yogurt[MeSH Terms]) OR (butter[MeSH Terms]) OR (cultured milk products[MeSH Terms]) OR (custard*[Title/Abstract]) OR (pudding*[Title/Abstract]) OR (cream*[Title/Abstract]) OR (cream[Title/Abstract]) OR (ice cream[Title/Abstract]) OR (ice-cream[Title/Abstract]) OR (curd*[Title/Abstract]) OR (porridge[Title/Abstract])

#2 (diet[Title/Abstract]) OR (diets[Title/Abstract]) OR (dietary[Title/Abstract]) OR (intake*[Title/Abstract]) OR (suppl*[Title/Abstract]) OR (consumption[Title/Abstract]) OR (food*[Title/Abstract]) OR (drink*[Title/Abstract]) OR (meal[Title/Abstract]) OR (nutrition[Title/Abstract]) OR (nutrient*[Title/Abstract]) OR (products[Title/Abstract])

#3 [(#1 AND #2]

Action 2 Outcome

#4 (Asthma[Title/Abstract]) OR (Asthma[MesH Terms]) OR (Asthma*[Title/Abstract]) OR (bronchoconstrict*[Title/Abstract]) OR (bronchial spasm*[Title/Abstract]) OR (bronchospasm*[Title/Abstract]) OR (bronch*[Title/Abstract])

Action 3 Combine exposure and outcome

#5 (#3 AND #4)

Action 4 Limits

#6 (Rats[Mesh: NoExp]) OR (Mice[Mesh: NoExp]) OR (rat[Title/Abstract]) OR (rats[Title/Abstract]) OR (mouse[Title/Abstract]) OR (mice[Title/Abstract]) OR (vivo[Title/Abstract]) OR (vitro[Title/Abstract])

#7 (#5 NOT #6)

Embase (<http://www.embase.com>)

Action 1 Determinants

#1 ('dairy':ab,ti) OR ('milk*':ab,ti) OR ('cheese*':ab,ti) OR ('yogurt*':ab,ti) OR ('yogurt*':ab,ti) OR ('butter':ab,ti) OR ('buttermilk':ab,ti) OR ('dietary pattern*':ab,ti) OR ('dairy products':ab,ti) OR ('milk':ab,ti) OR ('cheese':ab,ti) OR ('cultured milk products':ab,ti) OR ('custard*':ab,ti) OR ('pudding*':ab,ti) OR ('cream*':ab,ti) OR ('cream':ab,ti) OR ('ice cream':ab,ti) OR ('ice-cream':ab,ti) OR ('curd*':ab,ti) OR ('porridge':ab,ti)

#2 ('diet':ab,ti) OR ('diets':ab,ti) OR ('dietary':ab,ti) OR ('intake*':ab,ti) OR ('suppl*':ab,ti) OR ('consumption':ab,ti) OR ('food*':ab,ti) OR ('drink*':ab,ti) OR ('meal':ab,ti) OR ('nutrition':ab,ti) OR ('nutrient*':ab,ti) OR ('products':ab,ti)

#3 [(#1 AND #2]

Action 2 Outcome

#4 ('Asthma':ab,ti) OR ('Asthma*':ab,ti) OR ('bronchoconstrict*':ab,ti) OR ('bronchial spasm*':ab,ti) OR ('bronchospasm*':ab,ti) OR ('bronch*':ab,ti)

Action 3 Combine exposure and outcome

#5 (#3 AND #4)

Action 4 Limits

#6 ('rat':ab,ti) OR ('rats':ab,ti) OR ('mouse':ab,ti) OR ('mice':ab,ti) OR ('vivo':ab,ti) OR ('vitro':ab,ti)

#7 (#5 NOT #6)

Web of Science

Action 1 Determinants

#1 TS='dairy' OR 'milk' OR 'cheese*' OR 'yogurt*' OR 'yogurt*' OR 'butter' OR 'buttermilk' OR 'dietary pattern*' OR 'dairy products' OR 'milk' OR 'cheese' OR 'yogurt' OR 'butter' OR 'cultured milk products' OR 'custard*' OR 'pudding*' OR 'cream*' OR 'cream' OR 'ice cream' OR 'ice-cream' OR 'curd*' OR 'porridge'

#2 TS='diet' OR 'diets' OR 'dietary' OR 'intake*' OR 'suppl*' OR 'consumption' OR 'food*' OR 'drink*' OR 'meal' OR 'nutrition' OR 'nutrient*' OR 'products'

#3 [(#1 AND #2]

Action 2 Outcome

#4 TS='Asthma' OR 'Asthma*' OR 'bronchoconstrict*' OR 'bronchial spasm*' OR 'bronchospasm*' OR 'bronch*'

Action 3 Combine exposure and outcome

#5 (#3 AND #4)

Action 4 Limits

#6 TS='Rats' OR 'Mice' OR 'rat' OR 'mouse' OR 'vivo' OR 'vitro'

#7 (#5 NOT #6)

**Supplemental Table S2. Characteristics of studies that reported the relationship between dairy products and risk of osteoporosis**

| **Author** | **Year** | **Country** | **Age range/ Mean age (year)** | **Gender** | **Study design** | **N** | **case** | **Comparison** | **aOR or aHR (95% CI)** | **Adjusted variables** | **Assessment of dairy products** | **Diagnosis** | **Study quality** |
| --- | --- | --- | --- | --- | --- | --- | --- | --- | --- | --- | --- | --- | --- |
| Hijazi | 2000 | Saudi Arabia | 12 | M/W | Case-control study | 316 | 114 | Milk 2-3 times/day vs.>3 times/day <2 times/day vs.>3 times/day | 1.51 (0.70-3.26) 2.40 (1.21-4.75) | Place of residence, Nationality, Sex, Mother’s education, Family history of asthma/allergy, Positive skin test | semi-quantitative food frequency questionnaire | ISAAC (“Have you had wheezing or whistling in the chest in the last 12 months?” and “Have you ever had asthma?”) | 7 |
| Riedler | 2001 | Austria, Germany, and Switzerland | 6-13 | M/W | Cross-sectional study | 812 | 37 | Farm milk consumption vs. no | 0.48 (0.21-1.10) | age, sex, study area, parental education, family history of asthma and hay fever, number of older siblings and farming status. | Self-designed questionnaire | ISAAC (“Have you had wheezing or whistling in the chest in the last 12 months?” and “Have you ever had asthma?”) | 7 |
| Wijga | 2003 | Netherlands | 0-3 | M/W | Cohort study | 2978 | 195 | Full cream milk daily vs. no  Milk products daily vs. no | 0.54 (0.34-0.88)  0.74 (0.53-1.03) | sex, birth weight, presence of older siblings (yes/no), parental allergy (having (had) asthma or having an inhalant allergy), maternal level of education, breast feeding for at least 8weeks (yes/no), and smoking in the homewhen the childwas 2 years old (at least once a week v less than once a week) | Self-designed questionnaire (ISAAC) | “asthma”, defined as ever having had a doctor’s diagnosis of asthma | 8 |
| Fussman | 2007 | Austria, Great Britain, and Germany | 0-3 | M/W | Cohort study | 696 | 36 | Milk consumption vs. no | 0.18 (0.06-0.49) | gender, parental history of atopy, passive smoking exposure, country of residence, and maternal education. | Self-desinged questionnaire | doctor-diagnosed asthma | 7 |
| Garcia-Marcos | 2007 | Spanish | 6-7 | M/W | Cross-sectional study | 15523 | NA | 1-2 times/week vs. no ≥3 times/week vs. no | current occasional asthma 0.58 (0.35-0.94) 0.70 (0.49-1.01) current severe asthma 0.65 (0.31-1.40) 0.50 (0.28-0.90) | sex, older and younger siblings, reported BMI, maternal smoking and level of exercise | Self-desinged questionnaire | ISAAC (‘Has your child had wheezing or whistling in the chest during the last 12 months?) | 7 |
| Mai | 2007 | Canada | 8-10 | M/W | Nested Case-control study | 723 | 246 | ≤2 times/week vs. >2 times/week | Girls: 1.97 (0.97-3.99) Boys: 0.52 (0.23-1.19) | overweight | Self-designed questionnaire | Canadian Asthma Consensus Guidelines （symptoms and variable airway obstruction） cough, wheeze, shortness of breath, and response to current medications | 5 |
| Garcia | 2008 | Colombia | 6-14 | M/W | Cross-sectional study | 3256 (6-7) 3829 (13-14) | NA | ≥3 times/week vs. ≤2 times/week | 6-7: 1.5 (1.0-2.2); 13-14: 0.8 (0.6-1.0) | Pulse consumption, Time watching television, Exposure to maternal or guardian smoking in the last 12 months, Exposure to maternal or guardian smoking in the first year of life, Use of acetaminophen in the first year of life, Use of acetaminophen in the last 12 months, Use of antibiotic in the first year of life, Maternal education, Traffic on the street of the household, Presence of a cat in home during the last 12 months, Presence of a dog in home during the first year of life | Self-desinged questionnaire | ISAAC (Have you ever had wheezing or whistling in the chest in the last 12 months? | 7 |
| Rodriguez-Rodriguez | 2010 | Spanish | 8-13 | M/W | Cross-sectional study | 638 | 28 | high dairy consumption vs. low (g per day) | 2.62 (0.66-10.44) | energy intake, fat intake, age, gender, body mass index, parental history of asthma, atopic eczema and allergic rhinitis and parental cigarette smoking. | Self-desinged questionnaire (3-day food records) | diagnosed with asthma by a doctor/ an episode of asthma or an asthma attack during the past 12 months/medications at some time in the previous 12 months | 7 |
| Yang | 2015 | China | 7-12 | M/W | Nested Case-control study | 642 | NA | High consumption of milk vs. low | 2.604 (1.569-4.322) | case/control and region variables | A food ingestion frequency questionnaire from EuroPrevall questionnaire | Doctor-diagnosed asthma/recurrent dyspnea, wheezing or cough episodes. | 6 |
| Feng | 2016 | China | 13-14 | M/W | Case-control study | 854 | 317 | ≥3 times/week vs. ≤2 times/week | 3.85 (2.60-5.69) | age, gender and region | Self-desinged questionnaire | ISAAC (‘Have you had wheezing in your chest when you breathe during the past 12 months?’ and ‘Have you ever been diagnosed with asthma by a doctor?) or ( coexistence of current wheeze and Airway Hyperresponsiveness) | 6 |
| Koivusaari | 2022 | Finnish | 0-5 | M/W | Cohort study | 3053 | 184 | Cow's milk products vs. no | 1.04 (1.00-1.08) | maternal and paternal asthma and allergic rhinitis, gestational age, sex, atopic eczema during the first 6 months, cow's milk allergy, and intake of energy | Self-desinged questionnaire (3-day food records) | ISAAC (doctor- diagnosed asthma plus either wheezing symptoms or use of asthma medication during the preceding 12 months.) | 8 |


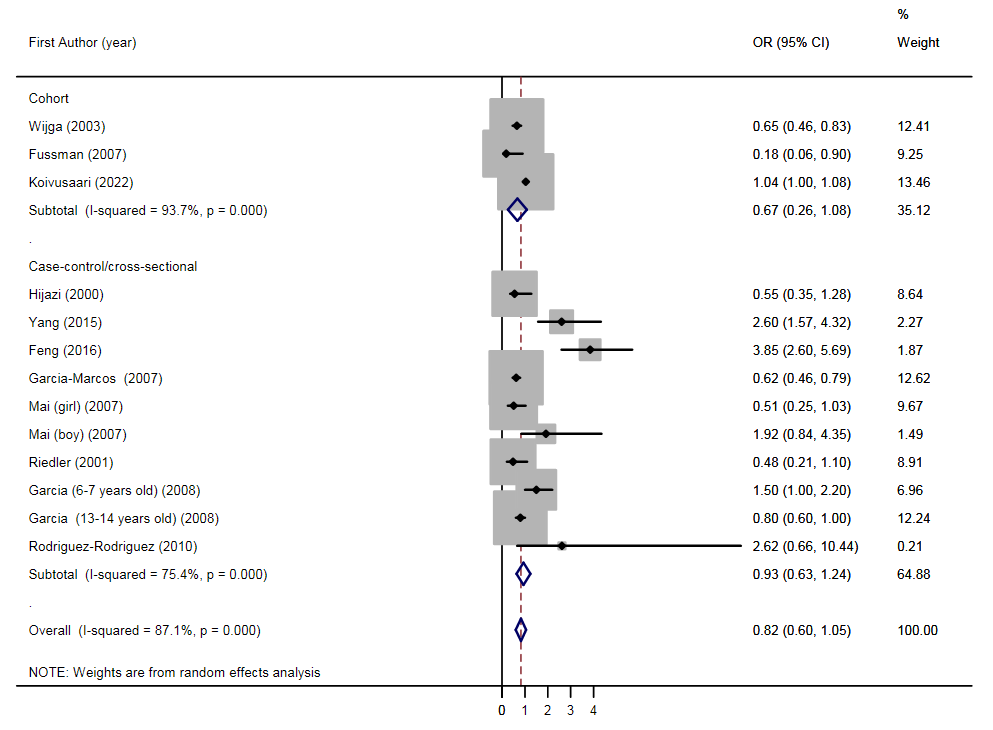

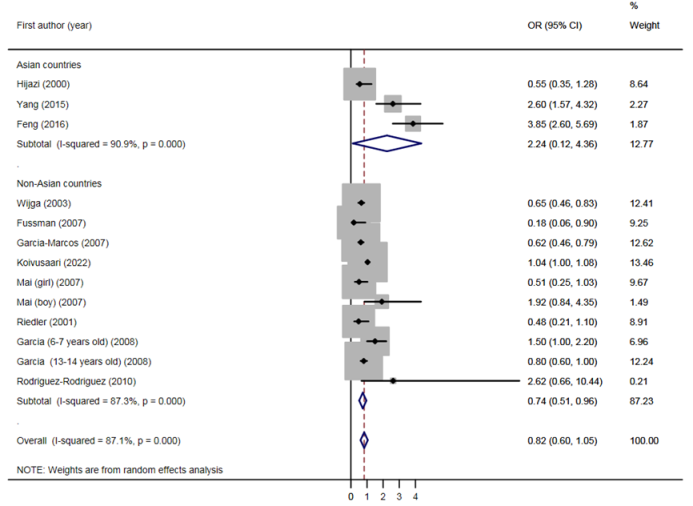

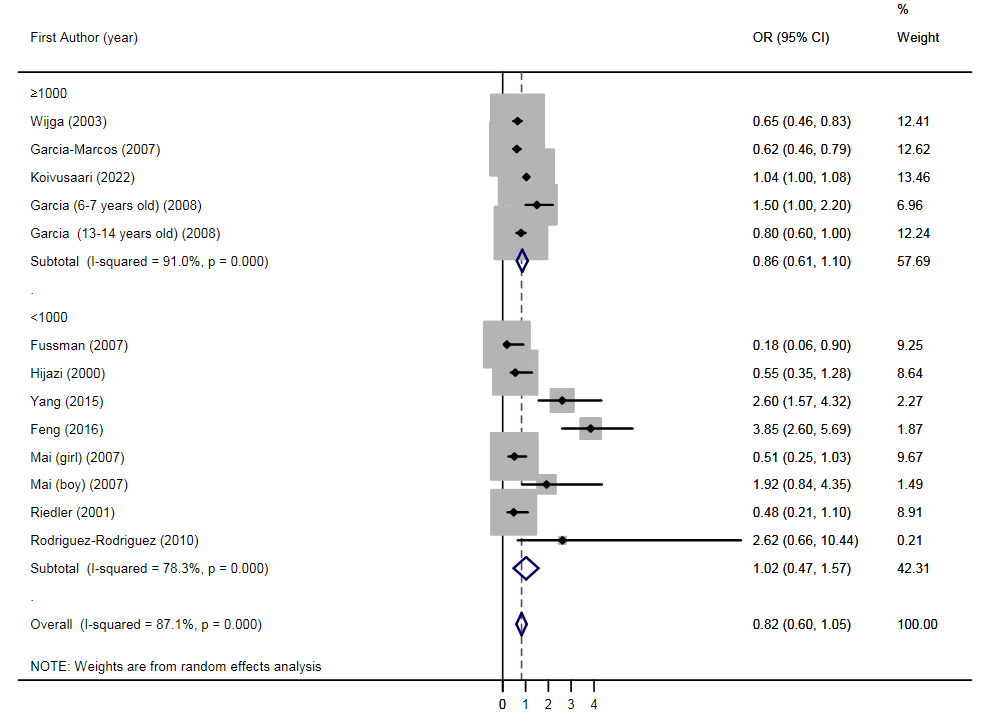


a (study design) b (Location) c (Sample size)


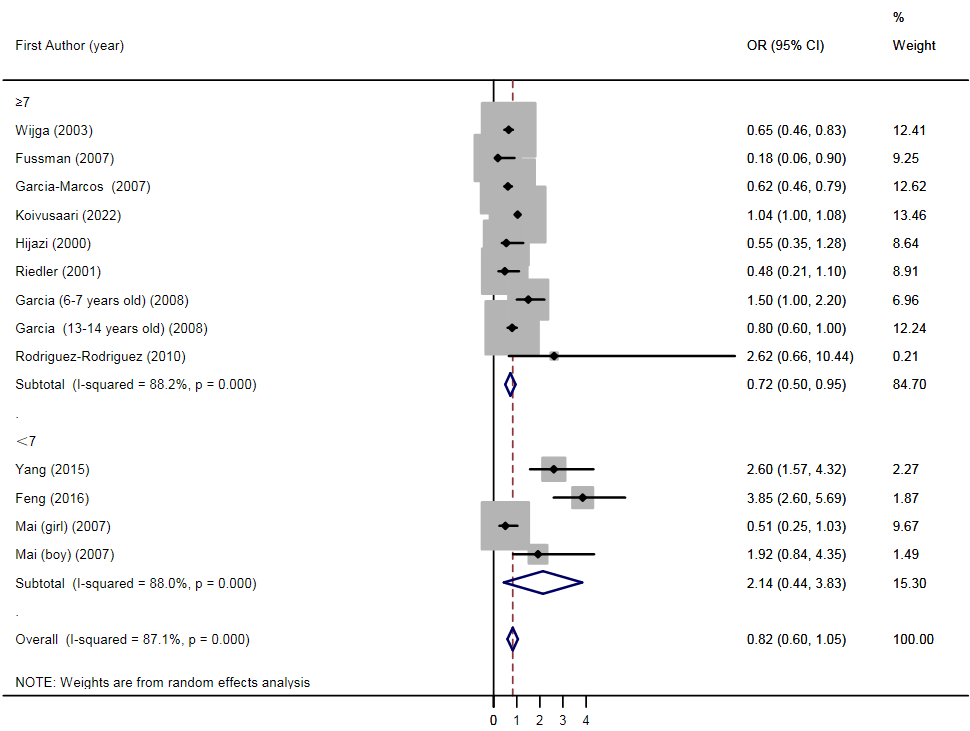

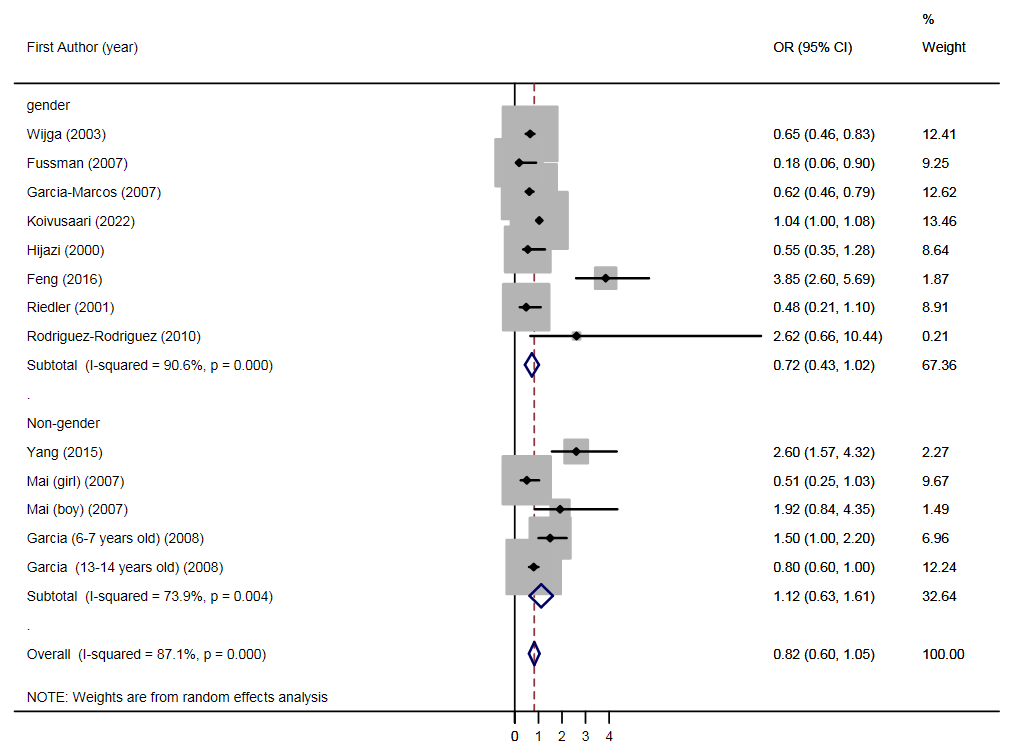

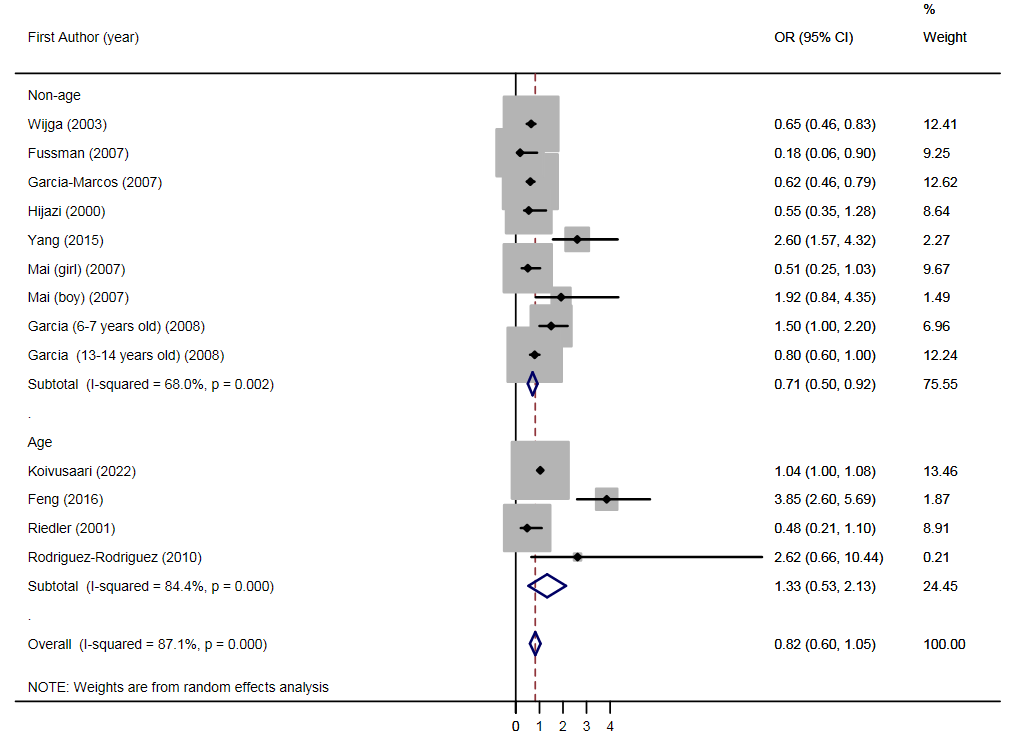


d (Quality score) e (adjustment for Gender) f (adjustment for age)

**Supplementary Figure S1. Forest plots of subgroup Analysis for milk and dairy consumption and risk of asthma**


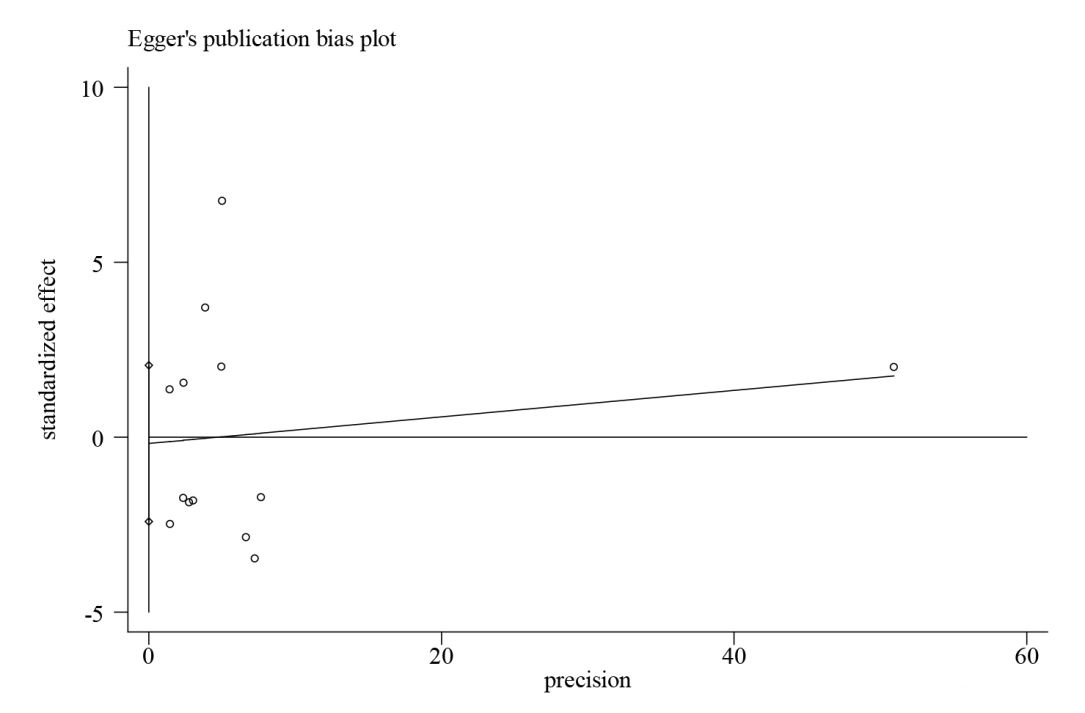


**Supplementary Figure S2. Egger’s plot of publication bias for the association of milk and dairy consumption and asthma**

| **Section and Topic** | **Item #** | **Checklist item** | **Location where item is reported** |
| --- | --- | --- | --- |
| **TITLE** | | |  |
| Title | 1 | Identify the report as a systematic review. | Line 1-2 |
| **ABSTRACT** | | |  |
| Abstract | 2 | See the PRISMA 2020 for Abstracts checklist. | Line 15-37 |
| **INTRODUCTION** | | |  |
| Rationale | 3 | Describe the rationale for the review in the context of existing knowledge. | Line 40-59 |
| Objectives | 4 | Provide an explicit statement of the objective(s) or question(s) the review addresses. | Line 59-61 |
| **METHODS** | | |  |
| Eligibility criteria | 5 | Specify the inclusion and exclusion criteria for the review and how studies were grouped for the syntheses. | Line 70-82 |
| Information sources | 6 | Specify all databases, registers, websites, organisations, reference lists and other sources searched or consulted to identify studies. Specify the date when each source was last searched or consulted. | Line 64-69 |
| Search strategy | 7 | Present the full search strategies for all databases, registers and websites, including any filters and limits used. | Line 67 |
| Selection process | 8 | Specify the methods used to decide whether a study met the inclusion criteria of the review, including how many reviewers screened each record and each report retrieved, whether they worked independently, and if applicable, details of automation tools used in the process. | Line 71-82 |
| Data collection process | 9 | Specify the methods used to collect data from reports, including how many reviewers collected data from each report, whether they worked independently, any processes for obtaining or confirming data from study investigators, and if applicable, details of automation tools used in the process. | Line 84-88 |
| Data items | 10a | List and define all outcomes for which data were sought. Specify whether all results that were compatible with each outcome domain in each study were sought (e.g. for all measures, time points, analyses), and if not, the methods used to decide which results to collect. | Line 85-88 |
|  | 10b | List and define all other variables for which data were sought (e.g. participant and intervention characteristics, funding sources). Describe any assumptions made about any missing or unclear information. | Line 85-88 |
| Study risk of bias assessment | 11 | Specify the methods used to assess risk of bias in the included studies, including details of the tool(s) used, how many reviewers assessed each study and whether they worked independently, and if applicable, details of automation tools used in the process. | Line 90-95 |
| Effect measures | 12 | Specify for each outcome the effect measure(s) (e.g. risk ratio, mean difference) used in the synthesis or presentation of results. | Line 97 |
| Synthesis methods | 13a | Describe the processes used to decide which studies were eligible for each synthesis (e.g. tabulating the study intervention characteristics and comparing against the planned groups for each synthesis (item #5)). | Line 103-104 |
|  | 13b | Describe any methods required to prepare the data for presentation or synthesis, such as handling of missing summary statistics, or data conversions. | Line 97-102 |
|  | 13c | Describe any methods used to tabulate or visually display results of individual studies and syntheses. | Line 104-105 |
|  | 13d | Describe any methods used to synthesize results and provide a rationale for the choice(s). If meta-analysis was performed, describe the model(s), method(s) to identify the presence and extent of statistical heterogeneity, and software package(s) used. | Line 103-115 |
|  | 13e | Describe any methods used to explore possible causes of heterogeneity among study results (e.g. subgroup analysis, meta-regression). | Line 109-111 |
|  | 13f | Describe any sensitivity analyses conducted to assess robustness of the synthesized results. | Line 109-111 |
| Reporting bias assessment | 14 | Describe any methods used to assess risk of bias due to missing results in a synthesis (arising from reporting biases). | Line 111-113 |
| Certainty assessment | 15 | Describe any methods used to assess certainty (or confidence) in the body of evidence for an outcome. | Line 109-111 |
| **RESULTS** | | |  |
| Study selection | 16a | Describe the results of the search and selection process, from the number of records identified in the search to the number of studies included in the review, ideally using a flow diagram. | Line 118-119; Figure 1 |
|  | 16b | Cite studies that might appear to meet the inclusion criteria, but which were excluded, and explain why they were excluded. | Figure 1 |
| Study characteristics | 17 | Cite each included study and present its characteristics. | Line 119-122 |
| Risk of bias in studies | 18 | Present assessments of risk of bias for each included study. | Supplementary Table S2 |
| Results of individual studies | 19 | For all outcomes, present, for each study: (a) summary statistics for each group (where appropriate) and (b) an effect estimate and its precision (e.g. confidence/credible interval), ideally using structured tables or plots. | Supplementary Table S2 |
| Results of syntheses | 20a | For each synthesis, briefly summarise the characteristics and risk of bias among contributing studies. | Figure 2 |
|  | 20b | Present results of all statistical syntheses conducted. If meta-analysis was done, present for each the summary estimate and its precision (e.g. confidence/credible interval) and measures of statistical heterogeneity. If comparing groups, describe the direction of the effect. | Line 124-135 |
|  | 20c | Present results of all investigations of possible causes of heterogeneity among study results. | Line 127-136 |
|  | 20d | Present results of all sensitivity analyses conducted to assess the robustness of the synthesized results. | Line 136-139 |
| Reporting biases | 21 | Present assessments of risk of bias due to missing results (arising from reporting biases) for each synthesis assessed. | Line 139-141 |
| Certainty of evidence | 22 | Present assessments of certainty (or confidence) in the body of evidence for each outcome assessed. | Line 130-135 |
| **DISCUSSION** | | |  |
| Discussion | 23a | Provide a general interpretation of the results in the context of other evidence. | Line 143-147 |
|  | 23b | Discuss any limitations of the evidence included in the review. | Line 196-198 |
|  | 23c | Discuss any limitations of the review processes used. | Line 189-195 |
|  | 23d | Discuss implications of the results for practice, policy, and future research. | Line 198-199 |
| **OTHER INFORMATION** | | |  |
| Registration and protocol | 24a | Provide registration information for the review, including register name and registration number, or state that the review was not registered. | We could not register the protocol of the review in the associated website, but our study was conducted based on the PRISMA statement. |
|  | 24b | Indicate where the review protocol can be accessed, or state that a protocol was not prepared. |  |
|  | 24c | Describe and explain any amendments to information provided at registration or in the protocol. |  |
| Support | 25 | Describe sources of financial or non-financial support for the review, and the role of the funders or sponsors in the review. | Line 225-230 |
| Competing interests | 26 | Declare any competing interests of review authors. | Line 217-218 |
| Availability of data, code and other materials | 27 | Report which of the following are publicly available and where they can be found: template data collection forms; data extracted from included studies; data used for all analyses; analytic code; any other materials used in the review. | Line 231-233 |

*From:*  Page MJ, McKenzie JE, Bossuyt PM, Boutron I, Hoffmann TC, Mulrow CD, et al. The PRISMA 2020 statement: an updated guideline for reporting systematic reviews. BMJ 2021;372:n71. doi: 10.1136/bmj.n71

For more information, visit: <http://www.prisma-statement.org/>
